# Disseminated Oligodendroglial-like Leptomeningeal Tumor of Childhood: A Distinctive Entity Revised and Correlated with Pathology

**DOI:** 10.5334/jbr-btr.1012

**Published:** 2017-04-25

**Authors:** Mooneera Peerboccus, Margarita Beltran-Marin, Eric Sariban, Quitterie Fontanges, France Ziereisen

**Affiliations:** 1 Radiology Dept, Université Libre de Bruxelles, Erasme Hospital, BE; 2 Radiology Dept, Erasme Hospital, BE; 3 Oncology Dept, Université Libre de Bruxelles, Queen Fabiola Children’s University Hospital, BE; 4 Anatomopathology Dept, Université Libre de Bruxelles, Erasme Hospital, BE; 5 Radiology Dept, Université Libre de Bruxelles, Queen Fabiola Children’s University Hospital, BE

**Keywords:** Leptomeninges, Low-grade, Pediatrics, Pseudocysts, Tumor

## Abstract

Disseminated oligodendroglial-like leptomeningeal tumor is a recently acknowledged entity whose radiological characteristics have rarely been discussed before. Typical of the childhood period, it should be differentiated clinically and radiographically from granulomatous or infectious conditions such as tuberculous meningitis, which is more common in this age group. The key to the diagnosis, even at an early stage, might be the presence of tiny T2 hyperintense lesions on the surface of the brain or spine. When suspected, a meningeal biopsy should be performed to confirm the diagnostic.

## Introduction

Disseminated oligodendroglial-like leptomeningeal tumors (DOLT), also called diffuse leptomeningeal oligodendrogliomatosis, are rare neoplasms with extensive dissemination in leptomeninges of brain and spine, with or without very small intraparenchymal lesions, most commonly depicted in the spinal cord [6]. This rare entity is typically seen in the pediatric population, with a median age of five years, and presents with a slowly progressive course [6], leading to a characteristic radiological profile. In this report, we present the radiologic findings and pathologic correlates of this condition as well as the key imaging findings that lead to the diagnostic, even at early stages of the disease.

## Case Report

**Figure 1:**
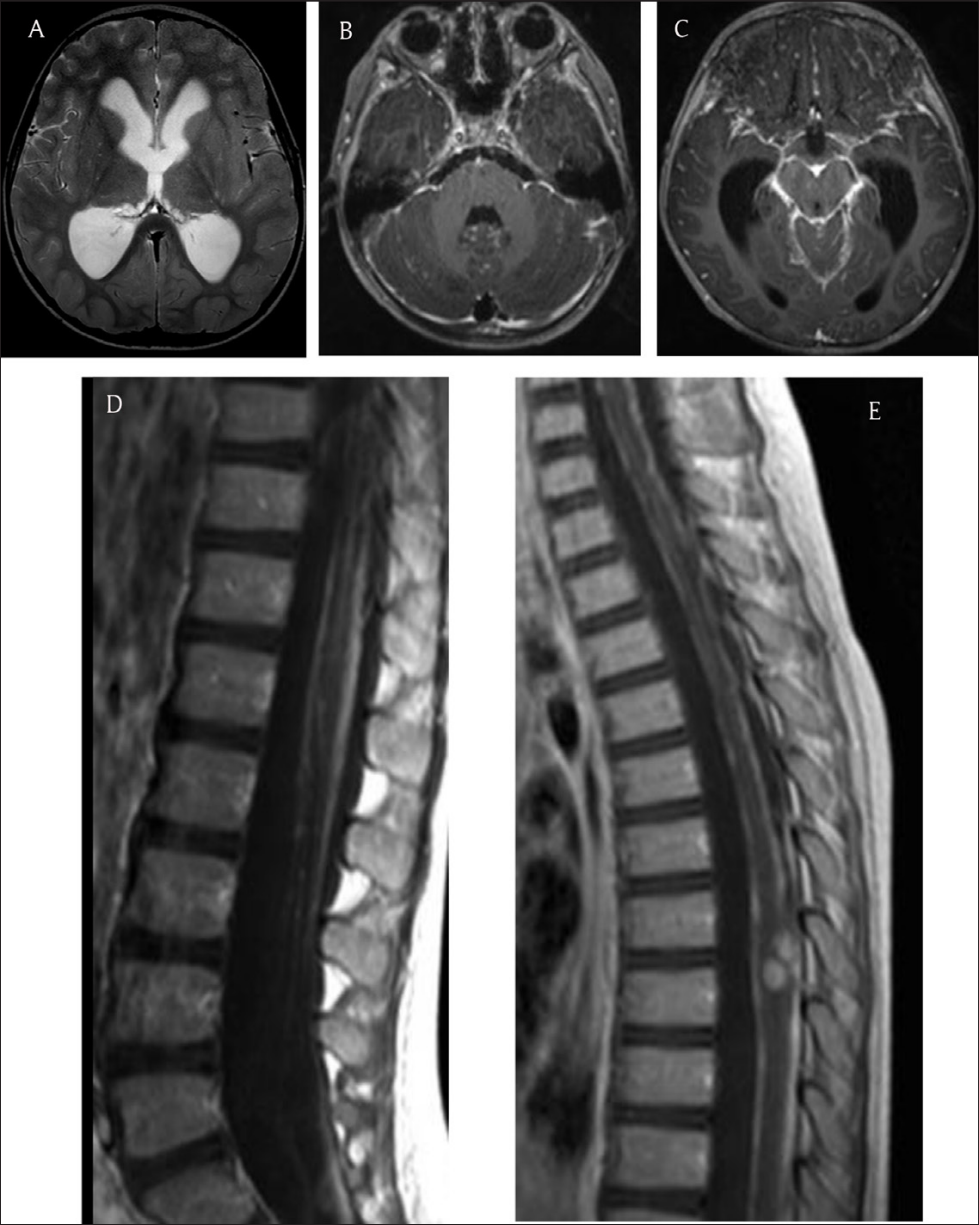
**(a)** Axial T2 image shows communicating hydrocephalus. Axial enhanced images **(b, c)** show leptomeningeal enhancement around the basilar region. Spine sagittal enhanced images show leptomeningeal enhancement around the roots of the cauda equina **(d)** and two intramedullary enhanced lesions **(e)**.

**Figure 2:**
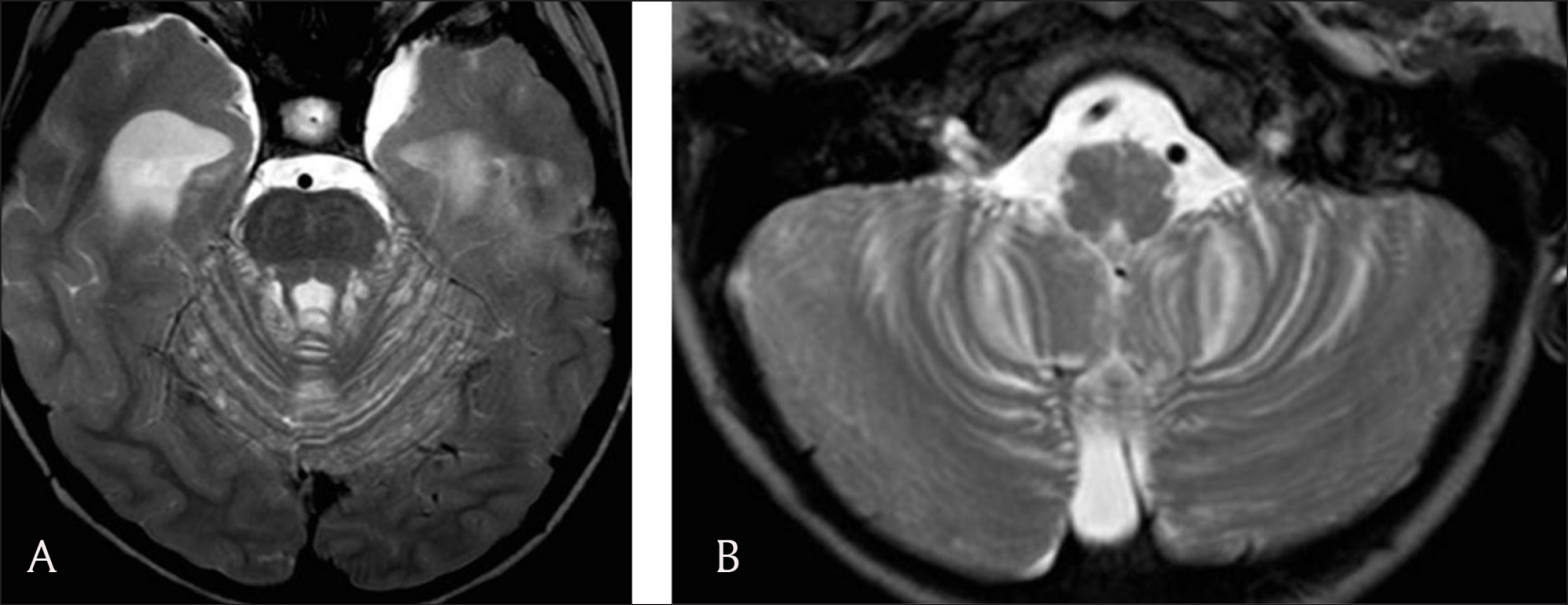
**(a, b)** Axial T2 images reveal pseudocystic lesions around the cerebellum and brainstem.

A four-year-old boy presented in October 2012 with a history of asthenia, vomiting, gait disturbance, and drowsiness lasting over two weeks. Neurologic examination revealed stiff neck, hyperreflexia in lower limbs, and moderate dysmetria. Blood samples showed no inflammatory syndrome. Brain and spine MRI demonstrated communicating hydrocephalus and diffuse leptomeningeal thickening and enhancement along the brain and spine, including few cranial nerves and nerve roots of the cauda equina (Figure 1a–d). Leptomeningeal enhancement was more pronounced in the basilar region. In addition, two intramedullary nodular enhanced lesions were observed at the level of the eighth and ninth thoracic vertebrae (Figure 1e). Moreover, tiny pseudocystic lesions, hyperintense on T2 and fluid-attenuated inversion recovery (FLAIR) images, showed up along the surface of the cerebellum, brainstem, thalamus, and tectal plate (Figure 2a, b). With an MRI simultaneously unveiling left apical lung condensation, the proposed diagnostic was diffuse meningitis, likely of tuberculous origin, with communicating hydrocephalus.

Repeated CSF analysis revealed slightly elevated proteins (82 mg/dl); bacteriology analysis was negative, and no neoplastic cells were found. PCR was positive for Mycobacterium tuberculosis. Intradermal test was negative.

The placement of a venticuloperitoneal shunt and anti-tuberculosis treatment (tetratherapy: Myambutol, Rifadin, Isoniazid, Tebrazid) associated with glucocorticoids resulted in a transient clinical improvement that lasted for two months. The lung lesion disappeared a month after the beginning of treatment. As from December 2012, the patient regularly suffered from early morning emesis, and in June 2013, he was admitted for neurological deterioration, including dysmetria, ataxia, and cranial nerve paresis associated with severe weight loss.

**Figure 3:**
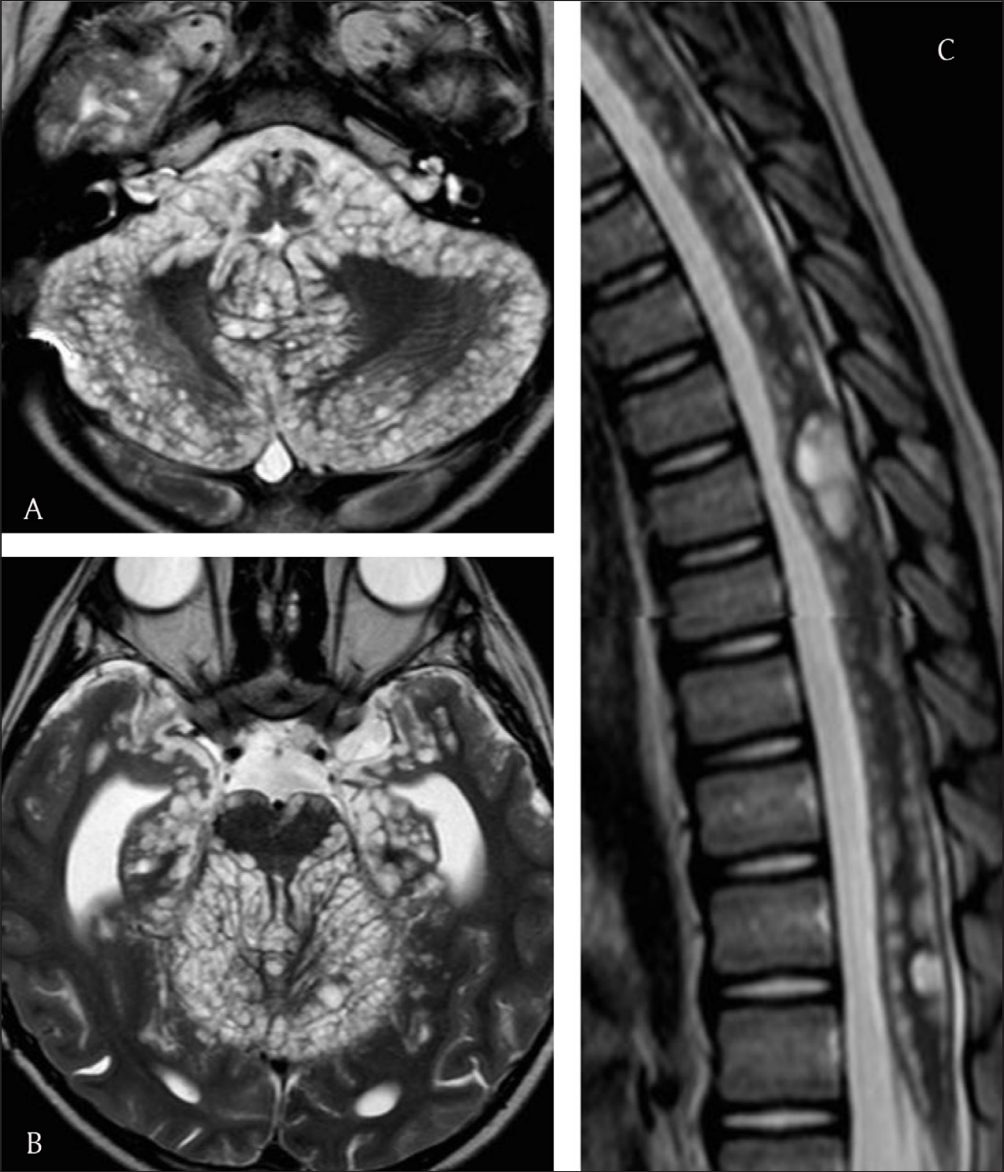
**(a–c)** Axial and sagittal T2 images depict a raise in the number and size of the pseudocystic lesions along the brain and spine.

He continued to clinically deteriorate despite a change in the anti-tuberculosis treatment and presented in May 2014 with repeated generalized seizures. MRI performed at that moment disclosed a raise in both the number and size of the small T2 hyperintense pseudocystic deposits, some of which were enhanced, along the parenchymal surface of the cerebellum, brainstem, anterior and medial temporal lobes, basal frontal lobes, thalami, and spinal cord (Figure 3a–c). The mass effect thereby entailed led to the worsening of the hydrocephalus and resulted into cerebellar herniation. Diffuse leptomeningeal enhancement persisted around the brain and spine. Moreover, a new focal, nodular intramedullary enhancement was observed in the spinal cord at the level of the first and second lumbar vertebrae.

**Figure 4:**
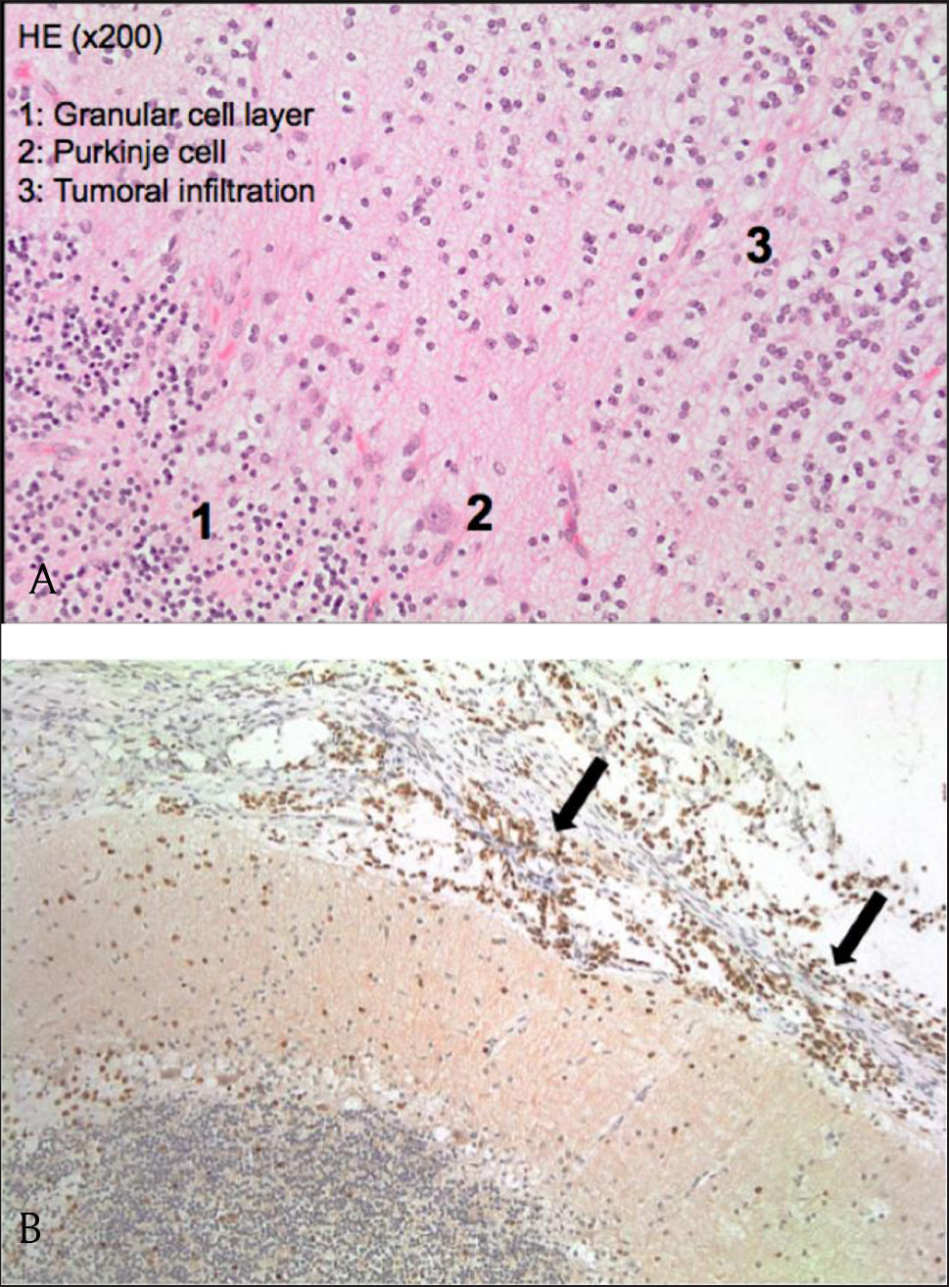
**(a)** Histopathology HE staining image shows cerebellar parenchymal tumoral infiltration. **(b)** Olig 2 immunostaining histopathology image (×100) shows leptomeningeal tumoral dissemination (arrows).

Surgery with decompressive craniectomy of the posterior fossa, along with biopsy of meninges, was performed. Histopathological examination showed a leptomeningeal infiltration by oligodendrocyte-like cells with rounded nuclei and perinuclear halo, showing positive immunostaining for OLIG2 and GFAP (Figure 4a, b). Growth fraction, evaluated using Ki 67 immunostaining, was 1–3 percent. Additional molecular biology analysis revealed chromosomic losses of 1p and 19q. The diagnostic retained was DOLT.

Chemotherapy (carboplatine and vincristine) was initiated, thereby helping to improve the patient’s clinical conditions. Follow-up MRI performed after 14 months of chemotherapy revealed no regression of the pseudocystic lesions along the surface of the brain and spine. Recent medical reports from October 2016 describe diffuse sensory-motor sequelae due to the diffuse leptomeningeal lesions as well as cerebellar disorders. Neurocognitive sequelae implied enrollment in a centre for children with special needs. Fits were also frequent due to an epileptogenic focus in the frontal lobe.

## Discussion

A disorder similar to that of our patient was first reported in 1942, and it was designated as “oligodendrogliomatosis of the cerebrospinal pathway” [2]. Afterwards, other reports have described similar conditions; however, it is not clear whether they constitute a single entity or rather a diverse group of neoplasms unified by diffuse leptomeningeal dissemination [6]. Diffuse leptomeningeal oligodendroglioma-like neoplasm is different from diffuse leptomeningeal gliomatosis, which is an invasion of the subarachnoid space or ventricular system by a primary intraparenchymal glioma and is much more common [3].

A recent paper defined diffuse leptomeningeal oligodendroglioma-like neoplasm as a desmoplasia inciting tumor, principally or exclusively involving leptomeninges and made up of low-grade-oligodendroglioma-like cells that are extensively disseminated in the subarachnoid space [6]. It typically affects children and young adults. A co-existing intramedullary mass is neither required nor exclusionary [6]. It has further been postulated that these tumors may arise from leptomeningeal heterotopias formed of small nests of glial tissue found within the subarachnoid space and present in around 1 percent of healthy individuals [1].

The neuroimaging profile is characteristic in advanced disease where innumerable, small pseudocystic implants are observed along the surface of the brain, mainly in the posterior fossa and spine, as reported in our case [5]. The challenge for radiologists is to be able to suggest the diagnostic during early stages of the disease, when hydrocephalus and diffuse leptomeningeal enhancement predominating in the basal cistern and spine are the main features. While diffuse leptomeningeal enhancement may be a feature of neurosarcoidosis, fungal meningitis, and secondary leptomeningeal carcinomatosis arising from primary brain tumors, the main differential diagnosis among immunocompetent patients is infectious meningitis mainly of tuberculous origin [7]. Clinical presentation is indeed similar and 80–90 percent of patients with tuberculous meningitis reveal communicating hydrocephalus and diffuse basal enhancement [4]. However, the presence of tiny pseudocystic lesions along the surface of the brain and spine is not usually described in infectious meningitis.

In our case, the clinico-radiological settings associated to an apical pulmonary condensation and a PCR positive for mycobacterium tuberculosis initially led to anti-tuberculous treatment, even though other infectious tests were negative. The clinical improvement yet observed was probably related to the implantation of a ventricular shunt.

To be able to suggest the diagnostic of DOLT early enough, the presence of pseudocystic T2 or FLAIR hyperintense lesions along the subpial surface of the spinal cord or the brain should be investigated. In our patient, those superficial pseudocystic lesions were seen in the initial MRI examination at the level of the cerebellum and brainstem.

From an anatomopathologic point of view, these T2 or FLAIR hyperintense pseudocystic lesions along the central nervous system surface actually correspond to tumor cell infiltration with focal rarefaction of surrounding neural tissue, the tumor implants being embedded in a myxoid stroma [4,6]. Microscopic evaluation further unveils a neoplastic population of cells morphologically consistent with oligodendrocytes. Molecular analysis may also demonstrate, as was the case for our patient, chromosomic 1p19q co-deletion, usually seen with oligodendroglial tumors in adults [6]. The tumor, which thus contains oligodendroglioma-like cells with low-mitotic activity, is considered a low-grade tumor [6]. Moreover, the glial or glioneuronal nature of the tumor can be specified through immunostaining techniques. These will reveal positivity for glial markers such as GFAP and OLIG2 as well as neuronal markers such as Synaptophysin or Neu N in case of a glioneuronal nature [5,6]. In our case, lesions were positive for OLIG2 and GFAP.

Widespread infiltration of basal cranial nerves by oligodendroglioma-like cells may be an additional feature that explains the enhancement along cranial nerves demonstrated in our patient [6]. The presence of an intraparenchymal lesion, usually in the spinal cord, should also be investigated, as it is found in 81 percent of reported cases [6]. Such lesions may be solid or cystic, and it is not clear whether they are formed primarily within the parenchyma. Actually, they may appear over the course of the disease [6]. One hypothesis suggests that they may represent the inward spread of leptomeningeal disease along Virchow-Robin spaces [5].

Clinical course is generally marked by periods of stability or slow progression, although evolution towards anaplasia and aggressiveness has been described in few cases [6]. Early treatment of this low-grade tumor is associated with a higher likelihood of durable stable disease, hence the reason for early diagnosis [6]. In our patient, although clinical conditions were clearly improved, MRI performed after 14 months of chemotherapy demonstrated no marked decrease in the pseudocystic lesions along the surface of the brain and spine. To our knowledge, this particularity has not previously been described.

Diagnostic can only be confirmed through biopsy, since cerebrospinal fluid frequently merely shows increased protein levels while more rarely revealing lymphocytosis [6]. Cytology is rarely positive; the absence of tumoral cells in the cerebrospinal fluid is explained by the entrapment of tumor cells by the desmoplastic tissue induced by the tumor [6].

To summarise, disseminated oligodendroglial–like leptomeningeal tumors are rare, low-grade, glial or glioneuronal tumors which predominate in the pediatric population. They are characterised by the presence of multiple, nodular cystic-like lesions scattered over the brain and spinal cord surface. Neuroradiologists and pediatric radiologists should be aware of the distinctive neuroimaging features of this entity and be able to recognise it even at an early stage. Indeed, this entity should not to be confused with an infectious or inflammatory condition, as early diagnosis is linked to better prognosis.
